# Effect of acute hyperthyroidism on blood flow, muscle oxygenation, and sympathetic nerve activity during dynamic handgrip

**DOI:** 10.1002/phy2.11

**Published:** 2013-06-12

**Authors:** Chester A Ray, Charity L Sauder, Dana M Ray, Yuichiro Nishida

**Affiliations:** Heart & Vascular Institute and the Department of Cellular & Molecular Physiology, General Clinical Research Center, Pennsylvania State University College of Medicine, Milton S. Hershey Medical CenterHershey, Pennsylvania, 17033

**Keywords:** Exercise, oxygen delivery, thyrotoxicosis

## Abstract

Hyperthyroidism induces marked changes in hemodynamics. Although considerable research has been done to study the effect of hyperthyroidism on the cardiovascular system, few studies have isolated the short-term, nongenomic effects of thyroid hormone on cardiovascular responses to exercise. We used near-infrared spectroscopy to measure muscle oxygenation, Doppler ultrasound to measure skeletal muscle blood flow, and microneurography to measure muscle sympathetic nerve activity (MSNA) during fatiguing dynamic handgrip in twelve healthy males (26 ± 1 years). Subjects were measured separately in both the euthyroid state, and acute hyperthyroid state (approximately ten times the normal levels of T_3_), induced by oral dosage of 300 μg of triiodothyronine (T_3_). Forearm blood flow was increased as a function of exercise time in the euthyroid and hyperthyroid state (Δ161.8 ± 45.0 mL/min and Δ140.7 ± 16.3 mL/min, respectively) but there was no significant difference between trials. Forearm vascular conductance (FVC) also increased as a function of exercise time with no significant difference between treatments at submaximal exercise but was significantly less with T_3_ treatment. MSNA was not different at rest or during submaximal exercise; however, MSNA was significantly greater at fatigue during the hyperthyroid state. Muscle oxyhemoglobin concentration was decreased during exercise in both euthyroid and hyperthyroid states (Δ19.7 ± 10.8% and Δ14.8 ± 9.6%, respectively); whereas deoxyhemoglobin concentration was increased (Δ50.0 ± 4.1% and Δ50.0 ± 6.2%, respectively). These results indicate that T_3_ had no direct effect on skeletal muscle oxygenation or blood flow during dynamic exercise, but elicited greater MSNA and lower FVC during fatiguing exercise.

## Introduction

Hyperthyroidism is associated with exercise intolerance, although the mechanism by which they are linked is still controversial (Martin et al. 1991; McAllister et al. 1995a; Kahaly et al. 1996). Studies have demonstrated that clinical hyperthyroidism is associated with increased heart rate and cardiac output, and decreased systemic vascular resistance (Forfar et al. 1982; Klein and Ojamaa 2001; Moolman 2002; Fazio et al. 2004). Despite this apparent hyperdynamic cardiovascular state in hyperthyroidism, dyspnea and exercise intolerance are common in hyperthyroid patients (Sestoft and Saltin 1988; Guleria et al. 1996; Kahaly et al. 1998; Irace et al. 2006). Prevailing theories on the mechanism for this phenomenon include down regulation of oxidative and glycolytic enzymes in skeletal muscle, remodeling of muscle tissue and loss of muscle mass, mitochondrial dysfunction, and altered cardiovascular function during exertion (Kahaly et al. 1996; Lombardi et al. 2002; Martin et al. 1991; McAllister et al. 1995a, 2000).

Many studies have focused on the long-term effects of hyperthyroidism present in patients with thyrotoxic disease, although few human studies have focused on short-term effects of thyroid hormone (Schmidt et al. 2002; Hiroi et al. 2006). Experimentally induced acute hyperthyroidism has the advantage of isolating nongenomic effects of thyroid hormone, and eliminates transcriptional regulation and muscle remodeling as potential confounding adaptations. Furthermore, while many cardiovascular variables have been studied in hyperthyroidism, no studies have monitored muscle oxygenation and blood flow to exercising skeletal muscle in acute hyperthyroidism in humans.

Because the cardiovascular system is altered in hyperthyroidism, and based on the fact that this system has a vital role in maintaining oxygenated blood flow to skeletal muscle during exercise, it is reasonable to speculate that these alterations may be mechanistically involved in hyperthyroid associated exercise intolerance. Studies have reported that hypoperfusion is not present in exercising skeletal muscle in hyperthyroid rats, but these studies have not been replicated in humans (McAllister et al. 1995b). It has been observed in humans that hyperthyroidism is associated with decreased oxygen uptake and delivery at the anaerobic threshold, although it is unclear what effect this has on muscle oxygenation during exercise (Kahaly et al. 1998). The purpose of this study was to monitor forearm muscle oxygenation by near-infrared spectroscopy (NIRS), blood flow by Doppler ultrasound, and muscle sympathetic nerve activity (MSNA) by microneurography in acute hyperthyroid subjects during dynamic forearm exercise. Based upon animal studies, we hypothesized that blood flow would increase to a greater degree in hyperthyroid versus euthyroid individuals during exercise. Furthermore, we hypothesized that muscle oxygenation would be lower in hyperthyroid than in euthyroid condition during exercise.

## Methods

### Subjects

This study was approved by the Institutional Review Board at the Penn State College of Medicine Milton S. Hershey Medical Center. Twelve healthy male volunteers (age 26 ± 1 years) were recruited and gave their written informed consent to participate in the study. Each subject was taking no medications at the time of the study and refrained from caffeine, exercise, and alcohol for 24 h prior to participating in the study.

### Study protocol

Venous blood samples were obtained before the study for measurement of plasma triiodothyronine (T_3_), via a brachial vein catheter. The blood sample was spun to separate the plasma. Plasma T_3_ was measured in duplicate by chemiluminescent microparticle immunoassay (Architect System, Abbott Laboratories, Abbott Park, IL). All samples were analyzed together after the completion of all tests. The lower limit of detection was ≤1.0 pg/mL. Subsequently, each subject was randomly given either a placebo or 300 μg oral liothyronine (Cytomel), a pharmacological preparation of triiodothyronine (T_3_), and then released from the lab for 6 h. Upon returning, blood was again drawn to assess thyroid hormone levels and the dynamic handgrip protocol was performed. During the placebo trial, a serum-free T_3_ level of 1.7–3.7 pg/mL was considered an acceptable range of normal thyroid status. After a period of 21–30 days, each subject returned to complete the study with the alternative treatment to serve as their own internal control. The study was designed as a double blind, crossover study.

### Dynamic handgrip

Subjects were monitored in the supine position. After a 5 min baseline measurement, subjects were asked to rhythmically grip and release against 1.1 kg of resistance for a total of 90 sec. Dynamic handgrip consisted of 1 sec of gripping followed by 1 sec of relaxation. After 15 sec rest, 1.1 additional kg of resistance was added and the 105 sec rest-gripping cycle was repeated. This process was continued until fatigue. Blood was drawn for analysis at baseline, after the first, third, fifth, seventh, and ninth additions of weight, and at fatigue and analyzed for lactate. The blood lactate levels were measured using Rapidlab 865 blood gas analyzer (Siemens, MA). In brief, lactate is oxidized by lactate oxidase to form hydrogen peroxide. The subsequent oxidation of hydrogen peroxide and the accompanying loss of electrons create a current flow that is directly proportional to the blood lactate concentration.

### Near-infrared spectroscopy

Near-infrared spectroscopy was used to monitor muscle oxygenation in the exercising forearm. Previous studies have demonstrated that hemoglobin absorbs light differentially in the range of 700–900 nm based on its oxygenation state (Wilson et al. 1989). Furthermore, it has been demonstrated that NIRS selectively measures oxygenation in microvasculature (less than 1 mm in diameter) and that at normal body temperatures, there is minimal contribution from skin blood flow (Mancini et al. 1994; Boushel et al. 1998; Davis et al. 2006).

Muscle oxygenation was monitored during dynamic handgrip using continuous wavelength NIRS (LEDI, Near Infrared Monitoring Inc, Philadelphia, PA) at 730 nm and 850 nm at a rate of 3 Hz. In addition, to ensure skin temperature did not elevate to a level that would interfere with our NIRS analysis, a skin temperature probe was taped over the dominant forearm and monitored at rest and during exercise (Serial Cable Systems, Henderson, NV).

A patch containing two light emitters and eight detectors was placed on the dominant forearm over the flexor digitorum profundus muscle and secured with a wrap bandage. Optical density (OD) at 730 nm was considered to be indicative of deoxygenated hemoglobin concentration and OD at 850 nm to be indicative of oxygenated hemoglobin concentration. Although it is not possible to calculate the actual concentrations of these molecules in tissue without knowing the path length of light in NIRS, a relative level of tissue oxygenation was sufficient for our purposes. We report results as OD and as a percent of the total labile signal (TLS). TLS defines complete oxygenation as the baseline OD prior to exercise, and complete deoxygenation as the OD during a 10 min arterial occlusion immediately following exercise by inflation of a pneumatic cuff around the arm to 220 mmHg. All experimental tissue oxyhemoglobin and deoxyhemoglobin concentrations are expressed as a percentage of this physiological range.

### Blood flow

Mean forearm blood velocity was recorded on a beat-by-beat basis using a 4-MHz pulsed Doppler ultrasound probe (model 500M Multigon, Yonkers, NY) with Zero Crossing (Hokanson, Bellevue, WA) during rest and dynamic handgrip. The probe was taped over the brachial artery proximal to the anticubital fossa on the dominant arm. The distance between the medial epicondyle of the humerus and the probe was the same during both visits. Likewise, focus, depth, power, and the gate of the probe were the same during both visits. Brachial arterial diameter measurements were taken at the end of diastole (determined by electrocardiogram) by measuring the distance between near and far wall intima–media using Doppler ultrasound (HDI 5000, ATL Ultrasound, Bothell, WA). Vessel diameters were averaged from a minimum of four heartbeats during baseline and at the end of each workload during the experimental protocol. Forearm arterial blood flow was calculated by multiplying the cross-sectional area (π*r*^2^) of the vessel by the mean blood velocity and by 60. The ratio of forearm blood flow and mean arterial blood pressure was used as an index of forearm vascular conductance (FVC).

### Muscle sympathetic nerve activity

Multifiber recordings of MSNA were obtained by inserting a tungsten microelectrode in the peroneal nerve of the leg. A reference electrode was inserted subcutaneously in close proximity to the recording electrode (Vallbo et al. 1979). The recording electrode was adjusted until a site with clear spontaneously occurring sympathetic bursts was established. Standard criteria for acceptable recordings of MSNA were applied. Raw nerve recordings were amplified (20,000–70,000 times), filtered (700–2000 Hz), full wave rectified, and integrated (0.1 sec time constant) to obtain mean voltage neurograms.

### Blood pressure and heart rate

Continuous measurements of mean arterial pressure and heart rate were made using a Finapres (Ohmeda, Louisville, CO). A three-lead system was used for recording an electrocardiogram during each study. All data were collected online (PowerLab 16sp, ADI Instruments, Newcastle, Australia) for later offline analysis.

### Statistical analysis

Results are expressed as mean ±SEM. Comparison between placebo and T_3_ treatments was performed using a two-within (treatment *x* exercise time) repeated measure ANOVA. *P-*value of <0.05 was considered statistically significant.

## Results

### Blood chemistry

All individual values were within our defined normal range for free T_3_ after placebo treatment (2.7 ± 0.1 pg/mL). Treatment with 300 μg T_3_ significantly raised serum-free T_3_ levels to 25.6 ± 1.2 pg/mL. Placebo treatment had no significant effect on free T_3_ levels.

**Figure 1:**
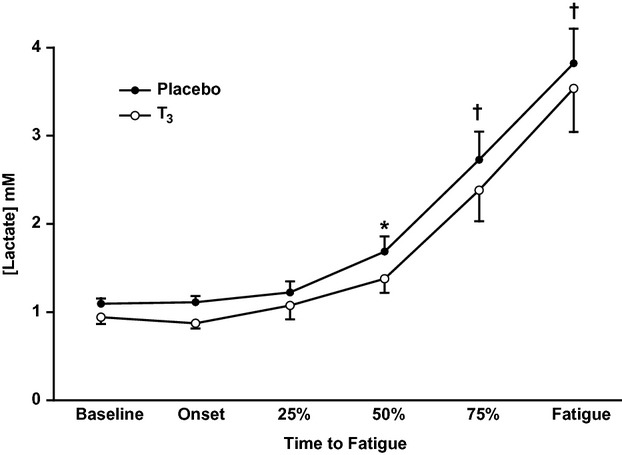
Blood lactate concentration during dynamic handgrip. No significant differences were found between treatments. *Significantly different from baseline for placebo only. ^†^Significantly different from baseline for both T_3_ and placebo, *P* < 0.05. Values are mean ± SE.

Blood lactate concentration in response to handgrip is summarized in Figure 1. Baseline lactate concentration was not significantly different between placebo (1.10 ± 0.06 mmol/L) and T_3_ treatment (0.94 ± 0.08 mmol/L). Lactate concentration increased as a function of exercise time to a maximum at fatigue in placebo (3.82 ± 0.39 mmol/L) and acute hyperthyroidism (3.54 ± 0.50 mmol/L). There was no significant difference between treatments.

### Exercise tolerance

Exercise tolerance was defined as the total amount of time from the start of dynamic handgrip to fatigue. We observed a slight increase in exercise tolerance in T_3_ treatment (1134 ± 36 sec) versus placebo (1066 ± 50 sec), which corresponds to approximately one additional workload (*P* = 0.07). There was no effect of familiarization with exercise time being the same for the first and second visits despite the treatment (*P* = 0.72).

### Heart rate and blood pressure

**Figure 2:**
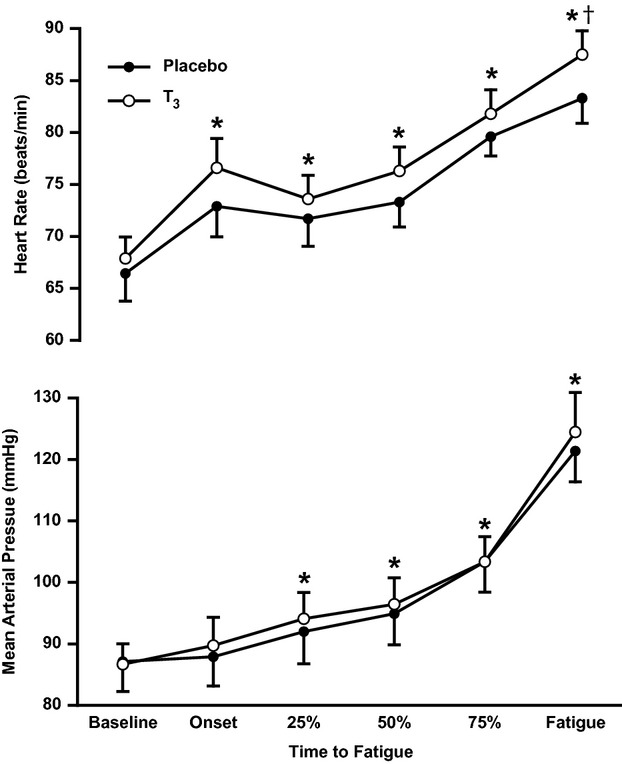
Heart rate and mean arterial pressure during dynamic handgrip. Heart rate was significantly elevated at fatigue in T_3_ compared with placebo but was not significantly elevated during submaximal exercise. *Significantly different from baseline for both T_3_ and placebo. ^†^Significantly different from placebo, *P* < 0.05.

Heart rate and mean arterial blood pressure responses to dynamic handgrip are presented in Figure 2. At baseline, there was no significant difference between placebo and T_3_ treatment (66 ± 3 and 68 ± 2 beats/min, respectively). Dynamic handgrip increased heart rate in both placebo and oral T_3_ treatment to 83 ± 2 and 88 ± 2 beats/min at fatigue, respectively. Heart rate was significantly elevated in acute hyperthyroidism versus placebo only at fatigue (Fig. 2). Dynamic handgrip raised mean arterial blood pressure (MAP) in both placebo and T_3_ treatment from 87 ± 5 mmHg and 87 ± 3 to 121 ± 5 mmHg and 124 ± 6 mmHg, respectively; but there was no significant difference in MAP response to exercise between treatments.

### Blood flow and vascular conductance

**Figure 3:**
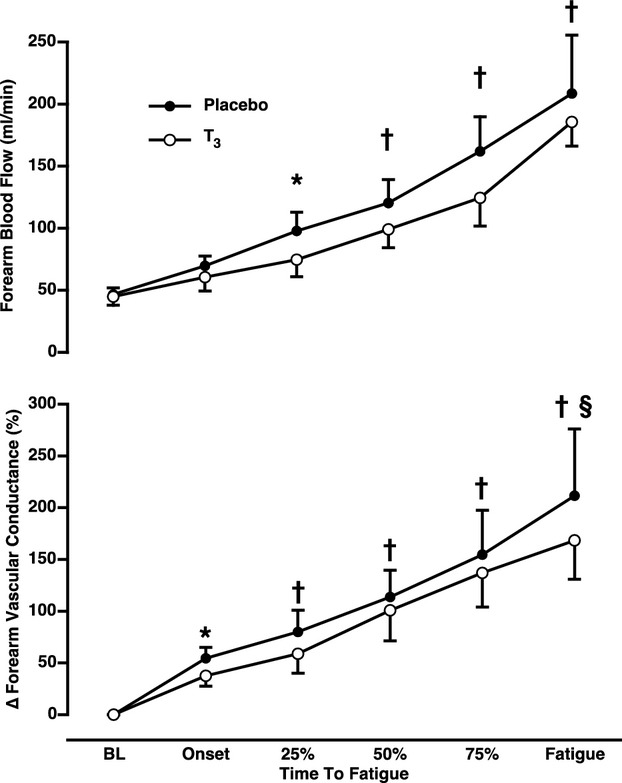
Blood flow and forearm vascular conductance during dynamic handgrip. No significant differences were found between treatments during submaximal exercise. Forearm vascular conductance was reduced at fatigue in the T_3_ trial compared with placebo. *Significantly different from baseline for placebo only. ^†^Significantly different from baseline for both T_3_ and placebo. ^§^Significantly different from T_3_, *P* < 0.05.

Data summarizing forearm blood flow at baseline and during dynamic handgrip exercise are presented in Figure 3. Blood flow at baseline was not significantly different between placebo and T_3_ treatments (46.6 ± 5.3 mL/min and 44.8 ± 6.9 mL/min, respectively). Blood flow increased as a function of exercise time to a maximum at fatigue in both placebo and T_3_ treatment (208.5 ± 47.0 mL/min and 185.5 ± 19.4 mL/min, respectively). However, no statistical significant difference in blood flow was noted between treatments.

Forearm vascular conductance responses to dynamic handgrip are presented in Figure 3. Results are reported as percent change from baseline in both placebo and T_3_ treatment. At fatigue, FVC increased in both placebo (212 ± 55%) and acute hyperthyroidism (168 ± 38%). Vascular conductance was significantly higher at fatigue in placebo than with T_3_ treatment (*P* = 0.046). There was no significant difference between treatments during submaximal exercise.

### Muscle sympathetic nerve activity

**Figure 4:**
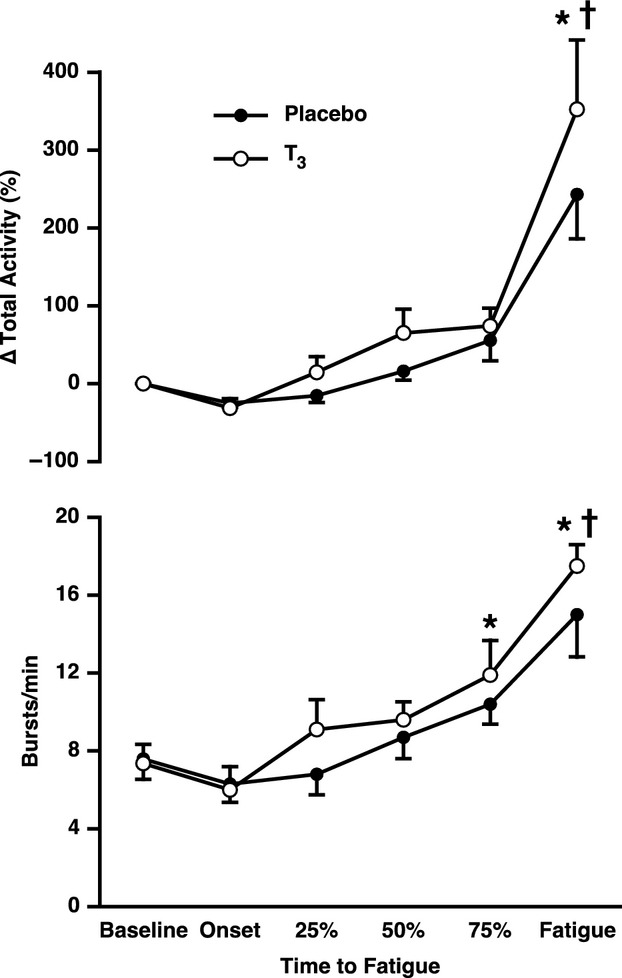
Muscle sympathetic nerve activity during dynamic handgrip. No significant differences were observed between treatments during submaximal exercise. MSNA was elevated in T_3_ compared with placebo at fatigue. *Significantly different from baseline for both T_3_ and placebo. ^†^Significantly different from placebo, *P* < 0.05.

Muscle sympathetic nerve activity at rest and during exercise is presented in Figure 4. At baseline, there was no difference in MSNA between placebo and T_3_ treatments. MSNA increased as a function of exercise time to a maximum at fatigue in both placebo (243 ± 57%) and T_3_ (352 ± 89%) treatments. MSNA was significantly elevated at fatigue in T_3_ versus placebo (*P* = 0.005) but not during submaximal exercise.

### Tissue oxygenation

**Figure 5:**
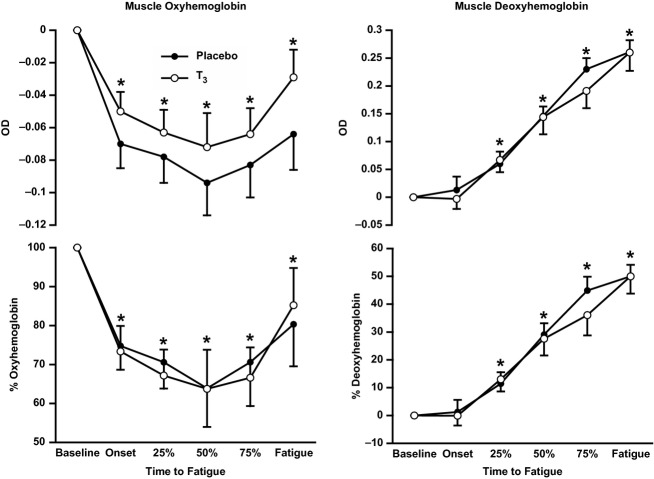
Muscle oxygenation by near-infrared spectroscopy. There was no significance of T_3_ treatment on muscle oxygenation during exercise. *Significantly different from baseline for both T_3_ and placebo.

Tissue oxygenation measured by NIRS is presented in Figure 5. Dynamic handgrip decreased oxyhemoglobin, defined by the concentration of oxygenated hemoglobin relative to muscle ischemia (0%) and baseline (100%), in both placebo and T_3_ treatments at fatigue (80.3 ± 10.8% and 85.2 ± 9.6% oxyhemoglobin, respectively; Fig. 5). No significant differences were found in muscle oxyhemoglobin concentration. Minimum tissue oxygenation occurred at 50% fatigue and was not significantly different in both placebo and T_3_ treatments (63.9 ± 9.9% and 63.7 ± 10.0% oxyhemoglobin, respectively). After 50% fatiguing exercise, tissue oxyhemoglobin concentration increased as a function of exercise time to fatigue (Fig. 4).

Dynamic handgrip to fatigue increased tissue deoxyhemoglobin, defined by the concentration of deoxygenated hemoglobin relative to baseline (0%) and muscle ischemia (100%), in both placebo and T_3_ treatments (50.0 ± 4.1% and 50.0 ± 6.2% deoxyhemoglobin, respectively). There was no significant difference in muscle deoxyhemoglobin between treatments. Baseline skin temperature was ∼30.0°C and increased slightly to 30.8°C at fatigue for both treatments.

## Discussion

To our knowledge, this is the first study that has monitored muscle blood flow, muscle oxygenation, and MSNA in response to exercise in acute hyperthyroidism in humans. The major new findings of this study are that acute elevation of circulating thyroid hormone concentration does not directly alter skeletal muscle blood flow or muscle oxygenation during dynamic handgrip in humans. Moreover, we did not observe significant differences in the cardiovascular or neural responses to submaximal exercise as a result of acute hyperthyroidism. However, acute elevation of T_3_ produced greater MSNA and lower FVC at fatigue. Our study design had the benefit of isolating direct effects of elevated T_3_ from the genomic effects of the hormone present in individuals suffering from chronic hyperthyroidism.

Although chronic hyperthyroidism is commonly associated with a hyperdynamic cardiovascular state, acute hyperthyroidism did not elicit these changes. The lack of an increase in forearm blood flow or vascular conduction in this study is in contrast to findings in animals by McAllister et al. (1995b) in long-term hyperthyroidism, and in contrast to studies by Park et al. (1997) and McAllister et al. (1995b) who demonstrated significant vasodilation in skeletal muscle in acute hyperthyroidism. McAllister et al. (1998) also reported reduced vascular contractile response to norepinephrine and enhanced acetylcholine-induced, endothelium-dependent vasorelaxation in isolated vessels from rats exposed to high levels of triiodothyronine for 6–12 weeks. However, all of these studies were performed in rats rather than humans, the latter in anesthetized rats, and may not be applicable to conscious humans. Our findings regarding forearm blood flow and vascular conduction are supported by MSNA. We observed no significant difference in the MSNA response to handgrip in the hyperthyroid compared to the euthyroid state during submaximal exercise. The data are consistent with findings in chronic hyperthyroid individuals in response to isometric handgrip exercise (Fagius et al. 1990). Our observation that heart rate was not significantly elevated at rest or during submaximal exercise in acute hyperthyroidism contrasts with findings in chronic hyperthyroidism which is typically elevated at rest and submaximal exercise (Forfar et al. 1982; Martin et al. 1991; Valcavi et al. 1992; Valensi et al. 1992). But no change in resting heart rate with T_3_ treatment is consistent with resting heart rate in short-duration hyperthyroidism (Schmidt et al. 2002).

During maximal exercise greater MSNA and lower FVC during T_3_ treatment was observed. Clearly the increased MSNA likely mediated the decline in FVC. The precise mechanism by which MSNA is increased by T_3_ at maximal exercise is unclear. This fact becomes even more complicated because MSNA is not affected during submaximal exercise and indicators of muscle oxygenation and metabolism were not affected.

Near-infrared spectroscopy was used to measure muscle oxygenation. NIRS has the advantageous ability to noninvasively measure muscle oxygenation without impairing normal muscle activity and allow real time measurements during exercise (Wilson et al. 1989; Chance et al. 1992). Furthermore, NIRS has been found to target changes in oxygenation of muscle microvasculature and minimally respond to changes in skin blood flow, as long as normal body temperatures are maintained (Mancini et al. 1994; Davis et al. 2006). Our experiments did not elicit substantial increases in forearm skin temperature during exercise as has been demonstrated before in a similar exercise model (Kondo et al. 2000), thus not invalidating the use of this technique to measure muscle oxygenation.

Although it is impossible to distinguish hemoglobin and myoglobin using NIRS due to their overlapping absorption spectra, it has been reported in several studies that less than 10% of the NIRS comes from myoglobin when measuring muscle oxygenation (Seiyama et al. 1988; Wilson et al. 1989; Chance et al. 1992; Wang et al. 2006). In contrast, studies have also suggested that myoglobin contributes significantly, or even primarily to NIRS signal (Hoofd 1999; Mole et al. 1999; Tran et al. 1999). Despite this controversy we chose to present our results as percent oxyhemoglobin and deoxyhemoglobin for the sake of simplicity, with the awareness that myoglobin may contribute to these values.

It has been suggested by Wang et al. (2006) that NIRS calculations of deoxyhemoglobin concentration are well correlated with O_2_ metabolic dynamics in exercising skeletal muscle. No differences were observed between treatments in deoxyhemoglobin concentration during exercise. In addition, we observed no significant difference in blood lactate levels between treatments in response to exercise. Because these variables were not altered by T_3_ treatment, our study indicates that thyroid hormone has no short-term effects on the metabolism of the exercising forearm muscle.

Our study was limited by examining cardiovascular variables in the forearm in response to exercise of a small muscle group. Schmidt et al. (2002) demonstrated a drop in systemic vascular resistance at rest in acutely hyperthyroid humans. It is possible that although blood flow and oxygenation of skeletal muscle was not affected, these variables may be affected in other organs in the acute hyperthyroid state. Furthermore, it is possible that these variables are altered during whole-body exercise. There are presently no published human studies examining cardiovascular variables in response to whole-body exercise in acute hyperthyroidism.

It is worth noting that the dose of T_3_ administered in this study achieved blood concentrations that were high compared to physiological levels of the hormone in patients suffering from clinical hyperthyroidism. If T_3_ does have a direct effect on forearm muscle oxygenation or blood flow at higher concentrations, it is unlikely to be physiologically relevant.

In summary, this study indicates that thyroid hormone has no direct effect on skeletal muscle oxygenation, blood flow, and blood lactate during dynamic forearm exercise, suggesting no significant changes in muscle oxygen delivery or metabolism. T_3_ itself does not appear to induce significant changes in hemodynamics at rest or in response to graded dynamic submaximal exercise; but it does mediate greater MSNA at fatigue. The hyperdynamic cardiovascular state commonly observed in clinical hyperthyroidism is likely due to long-term genomic effects of the hormone. The direct effects of thyroid hormone do not appear responsible for hyperthyroid-associated exercise intolerance.
